# Corrective Osteotomy in a Patient With Congenital Absence of Pronation Based on Three-Dimensional Statistical Shape Modeling

**DOI:** 10.1177/15589447231209343

**Published:** 2023-11-09

**Authors:** Eline M. van Es, Filip Stockmans, Joost W. Colaris

**Affiliations:** 1 Department of Orthopedics and Sports Medicine, Erasmus University Medical Center, Rotterdam, The Netherlands; 2 Department of Development and Regeneration, University of Leuven, Campus Kortrijk, Belgium

**Keywords:** corrective osteotomy, statistical shape modeling, congenital deformity, forearm rotation, patient-specific guides, three-dimensional

## Abstract

We present a new indication of a three-dimensional statistical shape model (SSM): a patient with bilateral impaired forearm rotation due to a congenital variance in bone shape. A corrective osteotomy was planned and performed to best match the SSM created by computed tomography (CT) scans of 18 peers. Postoperatively, pronation increased by 70°, and the patient was pain-free. A CT scan showed accurate correction of the deformity and union of all osteotomies. This technique offers opportunities for patients with bilateral nontraumatic osseous forearm pathology.

## Introduction

Nowadays, three-dimensional (3D) techniques are widely used for the planning of correction of malunited forearm bones.^1^ These 3D techniques use the mirrored unaffected side as a reference for planning a corrective osteotomy. However, in bilateral osseous forearm pathology, one cannot use the mirrored contralateral unaffected side as a template for correction. Previously, a 3D statistical shape model (SSM) was used to perform a corrective osteotomy in a patient with a posttraumatic malunion of both forearms.^2^ This innovative procedure resulted in malunion correction, improved forearm rotation, and pain relief.

Now we present a new indication for the use of an SSM: a patient with a rare bilateral congenital variance in bone shape resulting in impaired forearm rotation. Whereas a posttraumatic correction is reasonably predictable in terms of functional outcome, a congenital deformity is not. The bilateral deformity makes it even more challenging. The patient’s most deformed forearm had the most functional limitations, which increased our expectation that correcting this deformity could improve forearm rotation.

We aimed to investigate the possibility of performing a corrective osteotomy based on an SSM in a patient who had never been able to pronate and achieve functional improvement with this innovative technique.

## Methods

**Figure 1.**
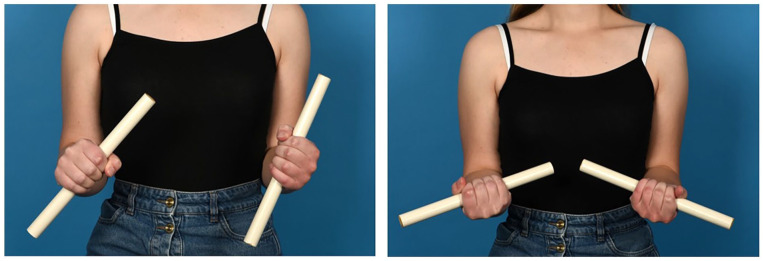
Patient with both forearms in maximum pronation (left) and maximum supination (right) before the surgery.

**Figure 2.**
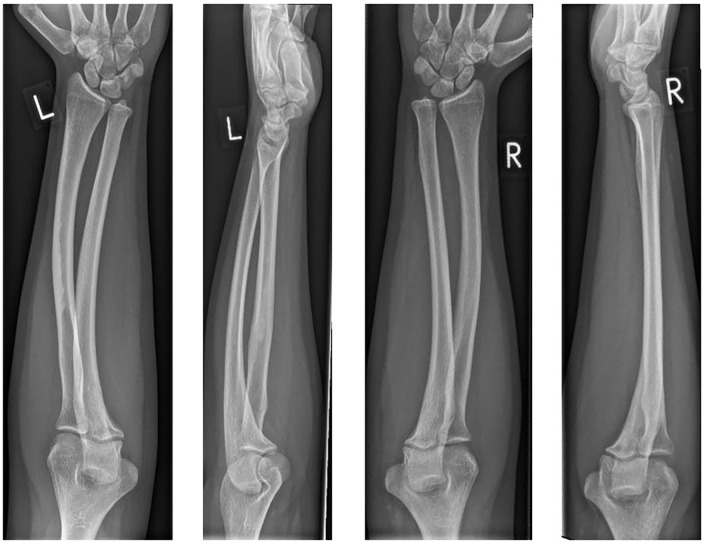
Posteroanterior and lateral radiographs of the left and right forearms (left to right) before the surgery.

**Figure 3.**
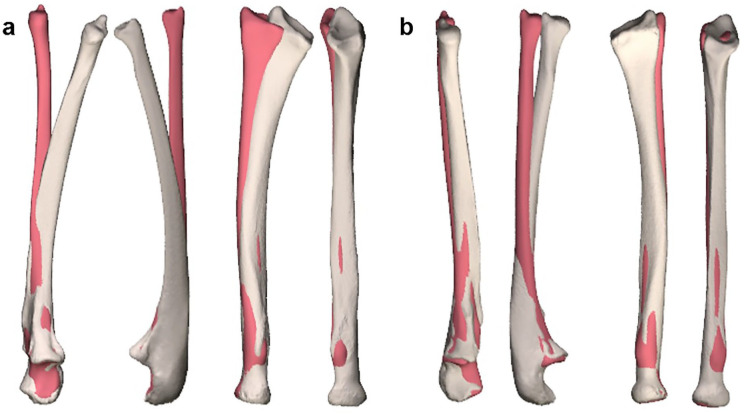
Coronal and sagittal view of the (a) left and (b) right ulna and radius. Ivory-colored bones show the radius and ulna of the patient. Red-colored bones show the statistical shape model proximally aligned with the patient’s bones.

An 18-year-old female with no history of trauma presented with negative pronation (−20°) of her left forearm and limited pronation (50°) of her right forearm. Supination was normal by 90° (Figure 1). Neither the patient nor her parents could remember having normal forearm rotation during childhood. Intensive physical therapy and static pronation braces did not result in an improvement in pronation. Radiographs and computed tomography scan–based 3D bone models (Mimics software, Materialise NV, Leuven) showed increased malalignment of particularly the left, but to a lesser extent also of the right radius and ulna (Figures 2 and 3). A magnetic resonance imaging scan showed no abnormalities, particularly in pronation and supination musculature, nor in the interosseous membrane or joint capsules. An evaluation under general anesthesia confirmed that no passive pronation on the left side was possible. The patient scored 4 points on the numeric rating scale (NRS) for pain during activity. Her main problem was using her arm, especially on the left, during her daily life activities and hobbies (typing, playing piano).

We used the Stryker Orthopedics Modeling and Analytics system (SOMA; Stryker, Kalamazoo, Michigan), which requires data sets from a minimum of 12 individual bone models to build a reliable SSM.^3^ We applied SOMA on 3D bone models of 18 individual forearms with a mean age of 15 years (range 10-23) and the gender distribution of 9 men and 9 women to create a template. We compared the 3D bone models of the patient’s forearm to the SSM. When aligned to the proximal end, the left arm with the most significant pronation limitation deviated the most from the model (Figure 3). This reinforced our hypothesis that a corrective osteotomy with the SSM as a template could improve pronation. The patient was informed about a corrective osteotomy based on an SSM, the possible risks and benefits, and the unsureness of the treatment outcome. She gave written informed consent for the procedure and for using her anonymized clinical information in publications.

We planned osteotomies of the left forearm to correct the deformity and realign both bones to match the SSM best. Correction of the ulna required a double osteotomy.

**Figure 4.**
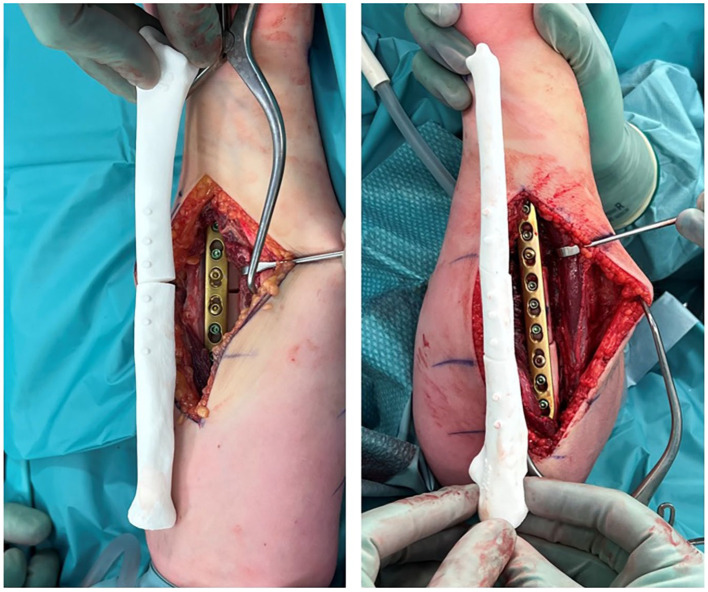
End-of-procedure pictures after plate fixation of the single osteotomy of the left radius and double osteotomy of the left ulna. The comparison with the three-dimensionally printed postoperative bone model is shown.

The preplanned osteotomies were performed with the patient under general anesthesia, using patient-specific 3D-printed drilling and cutting guides. The radius was fixated with a 6-hole and the ulna with a 9-hole LCP 3.5 plate (DePuy-Synthes, West Chester, Pennsylvania; Figure 4). Postoperative management consisted of an above-elbow cast in maximum pronation for 2 weeks, followed by hand therapy and a dynamic pronation splint for the night.

## Results

**Figure 5.**
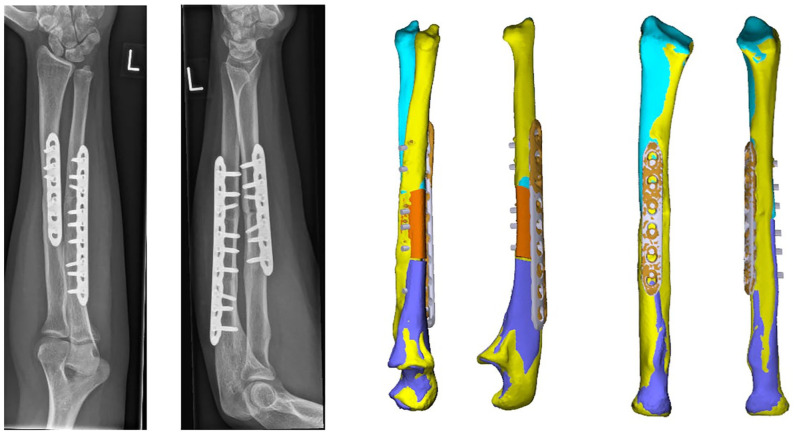
Posteroanterior and lateral radiographs and three-dimensional bone models with coronal and sagittal views of the left radius and ulna 1 year after surgery. *Note.* The planned distal part after the osteotomy is shown in blue, the middle part in orange (ulna only), and the proximal part in purple. Follow-up bones are superimposed in yellow. The planned position of the plate is shown in gray, and the actual position is in brown.

**Figure 6.**
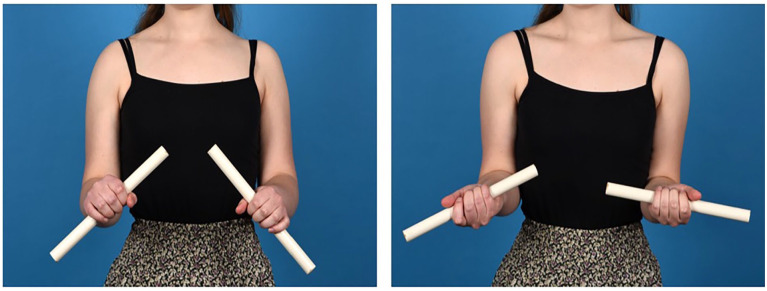
Patient with forearms in both maximum pronation (left) and maximum supination (right) 1 year after surgery.

Full passive pronation and supination were confirmed at the end of the procedure. The postoperative radiographs and 3D bone models showed accurate correction of both bones according to the 3D planning of the corrective osteotomy (Figure 5). Active forearm rotation improved gradually with hand therapy and a splint to 50° of pronation (70° improvement) 1 year after surgery. The patient had still full supination (Figure 6). She can better perform her daily activities and is very satisfied with the result. In addition, the patient is pain-free (NRS pain during activity = 0), and radiographs showed union of all osteotomies.

## Discussion

This case demonstrated a new indication for the innovative technique of correcting forearm deformities based on mimicking the alignment using SSM. We showed that a corrective osteotomy of both the radius and ulna in a patient with a congenital absence of pronation could result in a significant functional improvement.

Wang et al^4^ concluded in their systematic review that 3D shape prediction is a nascent but growing area of research. Most studies in their review aimed to simulate surgical outcomes based on computer models, but all were early-stage research and were some ways from clinical implementation.^4^ Mauler et al^5^ stated that an SSM could replace the contralateral-based planning by predicting the pretraumatic healthy shape of the pathological bone. Thereby, Oura et al^6^ also mentioned the potential for bilateral posttraumatic deformity correction by predicting the normal bone shape based on its partial shape. This technique was already successfully used in clinical practice as shown in an earlier case using an SSM in bilateral posttraumatic malunion with improved function.^2^

Although such a surgical procedure has never before been described in congenital pathology, the theoretical potential of this technique combined with the experience in a posttraumatic case encouraged us. This was reinforced by the fact that no cause other than bone shape was found and that the forearm that deviated most from the SSM had the least pronation.

This case extends the indication of correction of the forearm bones with the help of 3D-printed drilling and cutting guides from unilateral to bilateral posttraumatic malunions now to bilateral congenital deformities.
